# Green Tea Polyphenol Induces Changes in Cancer-Related Factors in an Animal Model of Bladder Cancer

**DOI:** 10.1371/journal.pone.0171091

**Published:** 2017-01-31

**Authors:** Tomohiro Matsuo, Yasuyoshi Miyata, Akihiro Asai, Yuji Sagara, Bungo Furusato, Junya Fukuoka, Hideki Sakai

**Affiliations:** 1 Department of Urology, Nagasaki University Graduate School of Biomedical Sciences, Nagasaki, Japan; 2 Department of Pathology, Nagasaki University Graduate School of Biomedical Sciences, Nagasaki, Japan; National Health Research Institutes, TAIWAN

## Abstract

Green tea polyphenol (GTP) suppresses carcinogenesis and aggressiveness in many types of malignancies including bladder cancer. However, the mechanistic basis of these effects is not well understood. This was investigated in the present study using a mouse model of chemically induced bladder cancer. C3H/He mice (8 weeks old; n = 46) were treated with 0.05% N-butyl-N-(4-hydroxybutyl) nitrosamine (BBN) solution for 14–24 weeks. Mice in the BBN + GTP group (n = 47) were also treated with 0.5% GTP solution over the same period. Tumor cell proliferation and microvessel density were evaluated along with immunohistochemical analysis of human antigen (Hu)R, vascular endothelial growth factor (VEGF)-A, cyclooxygenase (COX)-2, and hemeoxygenase (HO)-1 expression. Cytoplasmic HuR expression in cancer cells was higher at 14 and 24 weeks in the BBN than in the control group and was associated with increased invasion of tumor cells in muscle. However, these effects were not observed in the BBN + GTP group. A multivariate analysis of GTP intake and cytoplasmic HuR expression revealed that GTP was independently associated with COX-2 and HO-1 expression, while cytoplasmic HuR expression was associated with COX-2 and VEGF-A levels. Expression of COX-2 and HO-1 was associated with cell proliferation and that of VEGF-A and HO-1 was associated with angiogenesis. Nuclear HuR expression was not associated with any parameters such as carcinogenesis, muscle invasion, and GTP intake. These results indicate that GTP intake can suppress tumor progression and malignant behavior in an animal model of bladder cancer. We also speculate that GTP directly and indirectly suppresses tumor cell proliferation and angiogenesis via HuR-related pathways in bladder cancer.

## Introduction

Green tea is known to have health-promoting effects that are attributed to catechin polyphenols, which have anti-inflammatory and -oxidative properties [1, 2]. Many studies have demonstrated the anti-cancer effects of green tea polyphenol (GTP) in a variety of malignancies [3, 4, 5], and epidemiologic studies have shown that green tea consumption reduces cancer risk [6]. New cancer treatment strategies in combination with GTP intake have been recommended for several types of cancer [7, 8]. Thus, GTP is thought to be useful not only for cancer prevention but also for treatment.

The anti-cancer effects of GTP have been linked to the regulation of various cancer-related molecules [9, 10]. GTP also suppresses tumor growth and inhibits angiogenesis in cancer cells [11, 12], and was reported to have anti-tumorigenic effects in bladder cancer cell lines and an N-butyl-N-(4-hydroxybutyl)-nitrosamine (BBN)-induced mouse model of bladder cancer [13, 14, 15]. However, the mechanism underlying these effects is not well understood.

Bladder cancer is one of the most common cancers worldwide, with approximately one-quarter of patients progressing to an invasive stage of the disease [16]. Muscle invasion is known to be an initial step in metastasis and is a major cause of mortality in patients. Current treatments are unsatisfactory in terms of prolonging survival in the majority of patients with metastasis. As such, more effective treatment strategies are urgently needed. Green tea intake has been proposed as an option for preventing bladder cancer, although this is controversial [17], in part because it is difficult to establish effective GTP concentrations. In addition, other cancer-related external factors including smoking, exposure to chemical agents, and coffee intake must be considered [18, 19, 20].

To clarify the molecular mechanisms of GTP-induced anti-cancer effects, we used a mouse model of bladder cancer. We assessed tumor cell proliferation, microvessel density, and expression of human antigen (Hu)R, vascular endothelial growth factor (VEGF)-A, cyclooxygenase (COX)-2, and hemeoxygenase (HO)-1 for the following reasons: (1) HuR expression was suppressed by GTP in leukemia cells [21]; (2) HuR expression was positively associated with expression of VEGF-A, COX-2, and HO-1 [22–24]; and (3) these cancer-related molecules play important roles in malignant aggressiveness, including cell proliferation and angiogenesis in various types of cancer [25–27]. Our results indicate that GTP can effectively suppress bladder cancer growth and should therefore be considered as a preventative or therapeutic treatment approach for this disease.

## Materials and Methods

### Ethics statement

This study was approved by Institutional Animal Care and Use Committee (IACUC) of Nagasaki University (No: 0512210476). All animals were treated according to the Guidelines for Animal Experiments of Nagasaki University, and the study protocol was approved by the Regulations of Animal Care and Use Committee.

### BBN-induced bladder cancer model

BBN was obtained from Tokyo Kasei Industries (Tokyo, Japan). Extracted GTP (high-performance liquid chromatography, HPLC-grade GTP; ≥ 95% polyphenols) was purchased from LKT Laboratories (St. Paul, MN, USA; catalog no. G6817). Female C3H/He mice were obtained from Charles River Japan (Yokohama, Japan) at 6 weeks of age and housed in polycarbonate cages (two or three mice per cage) in a room with a controlled environment maintained at 22.8°C and 50% humidity on a 12:12-h light/dark cycle. The size of animal cage was 600.0 cm^2^ × 16.6 cm and mice were kept under pathogen-free conditions. Mice in the BBN group had unlimited access to 0.5% BBN in tap water starting from 8 weeks old for 14 or 24 weeks. In the GTP group, 0.5% GTP in tap water was administered in addition to BBN for similar periods. Normal controls received tap water only throughout the experiment. Food intake and body weight were recorded weekly and the health of the mice was monitored three times a week. Mice were scarified by cervical dislocation. In the present study, just one mouse in BBN group died without euthanasia at 3 weeks following the first BBN treatment. More details about the methods can be found in a previous report [15].

### Immunohistochemistry

The bladder was removed from euthanized mice, fixed in phosphate-buffered 10% formalin, embedded in paraffin, and cut into 5 μm-thick sections. In these fixed specimens, tumor volumes were calculated as (L × S^2^)/2, where L and S are the largest and smallest diameters, respectively, in millimeters. Immunohistochemical analysis was carried out as previously described [15]. Briefly, tissue sections were deparaffinized and rehydrated, and antigen retrieval was carried out at 95°C for 40 min in 0.01 M sodium citrate buffer (pH 6.0). Sections were immersed in 3% hydrogen peroxide for 30 min to quench endogenous peroxidase activity and then incubated with primary antibody overnight at 4°C. Immunoreactivity was visualized using a 3,3-diaminobenzidine tetrahydrochloride substrate kit (Zymed Laboratories, San Francisco, CA, USA) and sections were counterstained with hematoxylin. Detailed information on this procedure is provided in our previous reports [15, 27, 28].

### Evaluation

Microvessel density (MVD) was determined as the average number of cluster of differentiation 34-negative/von Willebrand factor-positive vessels in intra-tumoral areas in a high-power field (200×) [15]. Semi-quantificative analysis of HuR, VEGF-A, COX-2, and HO-1 signal intensity was performed based on an immunoreactivity score (IRS), where IRS = staining intensity × percentage of positive cells [29]. Staining intensity was scored as 0 (negative), 1 (weak), 2 (moderate), or 3 (strong). In addition, the extent of staining was scored as 0 (none), 1 (1%–20%), 2 (21%–50%), or 3 (51%–100%) according to the percentage of positive cells. All evaluations were carried out using a Nikon E-400 microscope (Tokyo, Japan) from digital images captured with a camera (Nikon DU100). A computer-aided image analysis system (Win ROOF v.5.0, Mitani, Fukui, Japan) was used to calculate various statistical variables. When the IRS of HuR, COX-2, VEGF-A, and HO-1 was above the third quartile of the interquartile range (IQR) in normal tissues, the tissue sample was judged as positive for cancer. Tumor volume, MVD, and proliferation index (PI) over the median value was judged as high.

### Statistical analysis

Data are expressed as median and IQR values. The Mann-Whitney U test was used to compare continuous variables and Scheffé’s method was used for multiple comparisons. The χ^2^ test and Fisher’s exact test were used for comparisons of categorical data. The Pearson correlation and correlation coefficient (r) were used to evaluate the relationship between continuous variables, and corresponding *P* values are shown. The crude and adjusted effects were estimated by logistic regression analysis. Variables that achieved statistical significance (*P* < 0.05) by univariate analysis were subjected to multivariate analysis [described as odds ratios (ORs) with 95% confidence intervals (CIs) along with *P* values]. Statistical analyses were carried out using StatView for Windows v.5.0 software (Abacus Concept, Berkeley, CA, USA).

## Results

### HuR expression is upregulated in bladder cancer

There was no difference in cancer frequency between the BBN (33.3%) and BBN+GTP (27.3%) groups at 14 weeks (*P* = 0.665; Table 1). During the same period, invasive cancer was detected in one mouse in each group. Likewise, at 24 weeks cancer incidence was similar between BBN (80.0%) and BBN + GTP (68.0%) groups (*P* = 0.333); however, the frequency of invasive cancer was lower in the BBN+GTP than in the BBN group (24.0% vs. 64.0%; *P* = 0.006).

**Table 1:** Frequencies of cancer according to green tea polyphenol intake.

	Cancer at 14 weeks			Cancer at 24 weeks		
	N	Entire	Invasive	N	Entire	Invasive
BBN	21	7 (33.3)	1 (4.8)	25	20 (80.0)	16 (64.0)
BBN+GTP	22	6 (27.3)	1 (4.5)	25	17 (68.0)	6 (24.0)
*P* value		0.665	0.906		0.333	0.006

BBN; N-butyl-N-(4-hydroxybutyl) nitrosamine, GTP; green tea polyphenol.

**Fig 1:**
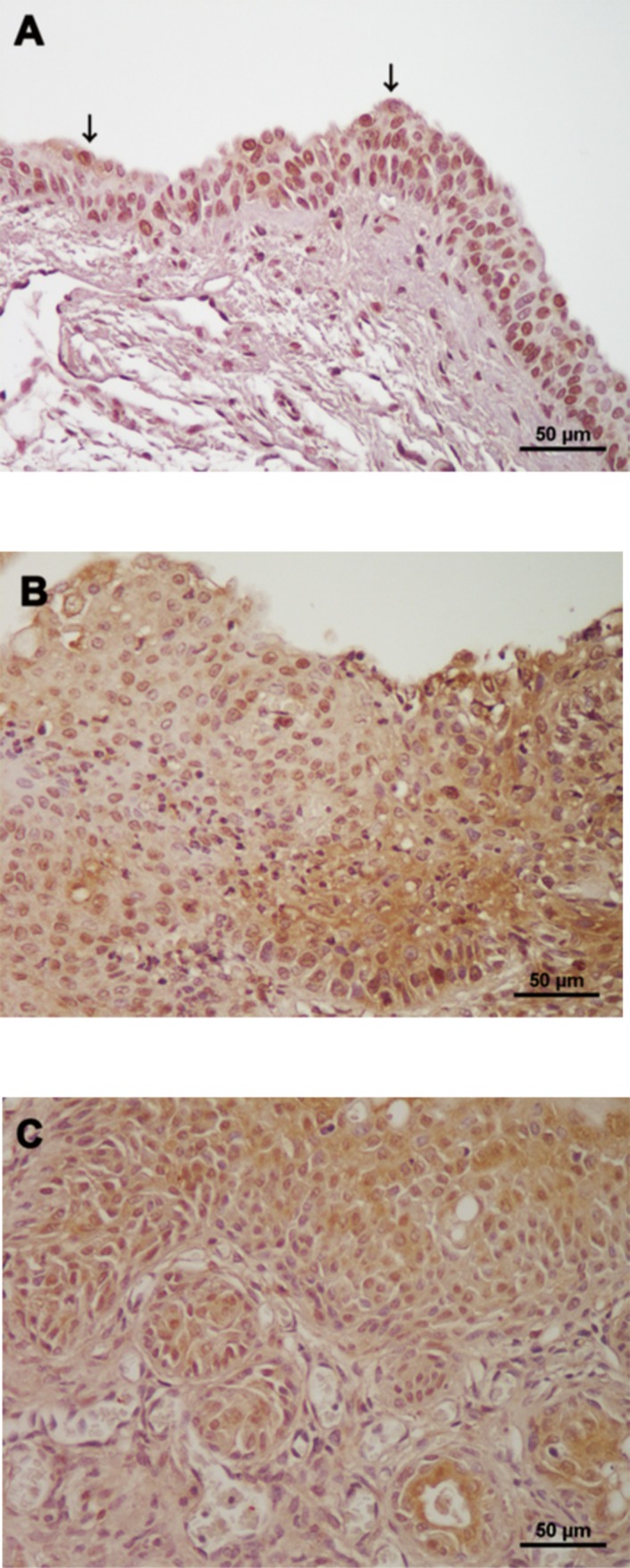
Representative examples of HuR immunoreactivity. HuR expression was observed in nuclei of both non-cancer (A) and cancer cells (B). In contrast, cytoplasmic HuR-positive non-cancer cells (arrows) were relatively rare (A). Although cytoplasmic expression of HuR was detected in bladder cancer cells (B and C), the percentage of cytoplasmic HuR-positive cancer cells in cases with muscle invasion (C) was higher than that in cases without muscle invasion (B).

**Fig 2:**
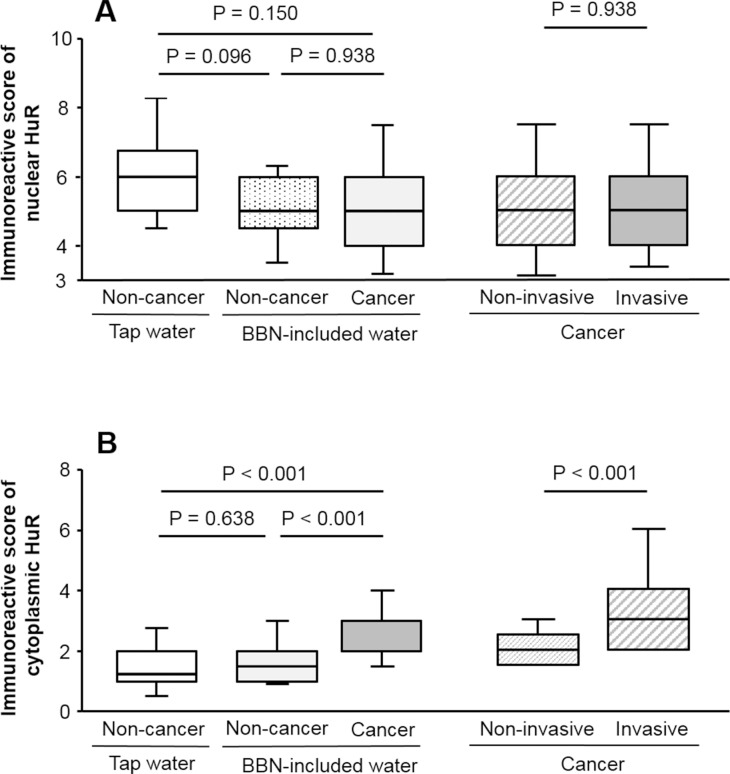
HuR expression in non-cancer and cancer cells. IRS of nuclear HuR differed between cancer and non-cancer cells and between invasive and non-invasive cancer cells (A). Cytoplasmic HuR expression and the IRS of cytoplasmic HuR was higher in cancer than in non-cancer cells, and higher in invasive as compared to non-invasive cases (B).

In non-cancer cells, HuR immunoreactivity was mostly observed in the nuclei (Fig 1A). In cancer cells, in addition to nuclei, HuR expression was detected in the cytoplasm (Fig 1B and 1C). Furthermore, we observed that the percentage of cytoplasmic HuR-positive cancer cells in cases of muscle invasion (C) was higher than that in cases without muscle invasion (B). In fact, the IRS of nuclear HuR in non-cancer cells was lower in mice that were administered BBN-containing water as compared to normal tap water, although the difference was not statistically significant (*P* = 0.096; Fig 2A). Likewise, there was no difference in the IRS of nuclear HuR between cancer and non-cancer cells in mice given BBN-containing water (*P* = 0.938; Fig 2A). In contrast, the IRS of cytoplasmic HuR was higher in cancer than in non-cancer cells (*P* < 0.001; Fig 2B) and also higher in invasive as compared to that in non-invasive cancer (*P* < 0.001), which was not true of nuclear HuR IRS (*P* = 0.938).

### GTP suppresses HuR expression in bladder cancer

The IRS of cytoplasmic HuR was similar between cancer and non-cancer tissues at 14 weeks (*P* = 0.391). However, an analysis with Scheffé’s method showed that the IRS was higher in the cancer group at 24 weeks relative to controls (*P* < 0.001) and relative to the value at 14 weeks (*P* = 0.004). On the other hand, the IRS of nuclear HuR did not differ between cancer and non-cancer groups at 14 weeks (*P* = 0.999) nor between the BBN groups at 14 and 24 weeks (*P* = 0.213), indicating that N-HuR expression was not correlated with pathological characteristics or GTP intake.

**Fig 3:**
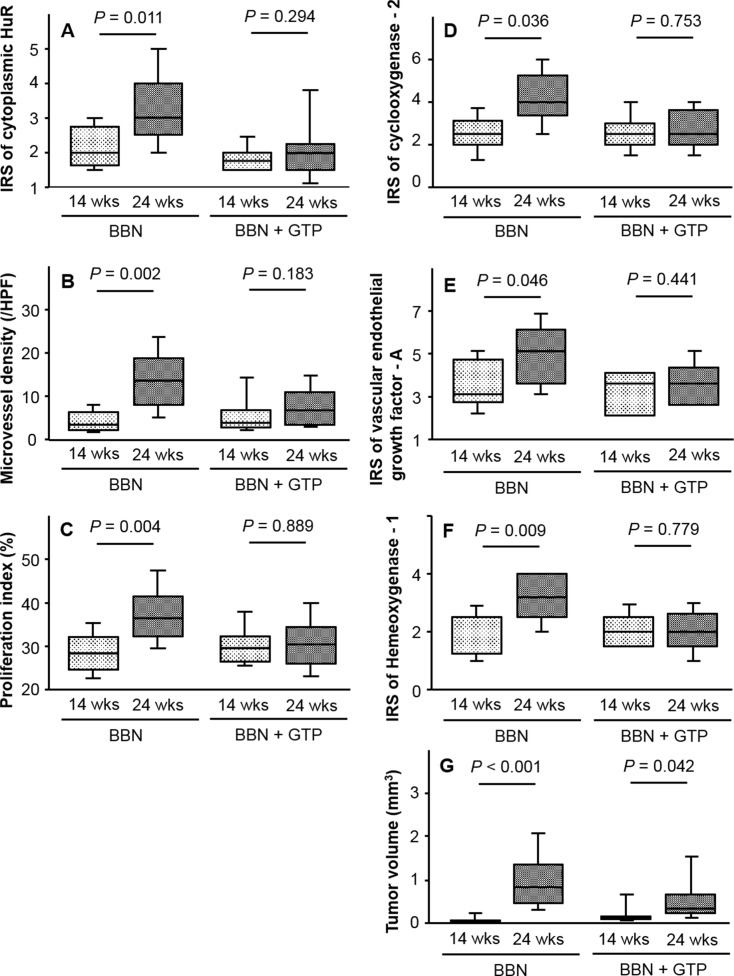
HuR expression and GTP intake. IRS of cytoplasmic HuR increased from 14 to 24 weeks in the BBN but not the BBN + GTP group (A). Similar trends were also observed for MVD (B), proliferation index (C), and COX-2 (D), VEGF-A (E), and HO-1 (F) expression. On the other hand, tumor volume increased in both groups during this period (G).

We next examined changes cytoplasmic HuR expression in response to GTP. The IRS of cytoplasmic HuR increased from 14 to 24 weeks in the BBN but not in the BBN + GTP group (*P* = 0.011 and 0.294, respectively; Fig 3A). Similar trends were observed for MVD (Fig 3B), PI (Fig 3C), and the IRS of COX-2 (Fig 3D), VEGF-A (Fig 3E), and HO-1 (Fig 3F). On the other hand, tumor volume increased in both BBN and BBN + GTP groups from 14 to 24 weeks (Fig 3G).

### GTP intake is associated with cancer-related factors

In univariate analyses, the IRS of cytoplasmic HuR was associated with all cancer-related factors examined. In similar analyses, GTP intake was associated with PI and the IRS of COX-2 and HO-1, as well as tumor growth, MVD, and PI when analyzed over a 24-week period (Table 2). Tumor invasion was associated with increased tumor size (OR = 84.33, 95% CI = 12.84–554.0, *P* < 0.001), high MVD (OR = 15.96, 95% CI = 3.99–63.85, *P* < 0.001), high PI (OR = 27.50, 95% CI = 6.06–124.8, *P* < 0.001), and upregulation of COX-2 (OR = 5.18, 95% CI = 1.48–18.19, *P* = 0.010), VEGF-A (OR = 15.96, 95% CI = 3.99–63.85, *P* < 0.001), and HO-1 (OR = 6.08, 95% CI = 1.72–21.50, *P* = 0.005) expression. A multivariate analysis revealed that cytoplasmic HuR expression was associated with the expression of COX-2 (OR = 1.52, 95% CI = 1.10–8.71, *P* = 0.033) and VEGF-A (OR = 2.83, 95% CI = 1.11–7.20, P = 0.029), while GTP intake was also associated with these factors (COX-2: OR = 0.21, 95% CI = 0.05–0.91, *P* = 0.037 and VEGF-A: OR = 2.83, 95% CI = 1.11–7.20, *P* = 0.029).

**Table 2:** Effect of green tea polyphenol intake on cytoplasmic HuR expression.

	Uni-variate analyses			Multi-variate analyses*		
	OR	95% CI	*P* value	OR	95% CI	*P* value
For large tumor						
GTP: intake	0.31	0.10–0.98	0.050			
GTP: 24 wks	22.2	2.48–190.0	0.005	23.1	0.84–634.1	0.063
C-HuR: positive	4.96	1.88–13.1	0.001	4.07	0.84–19.63	0.081
For high MVD						
GTP: intake	0.37	0.12–1.16	0.088			
GTP: 24 wks	8.07	1.56–41.7	0.013	3.04	0.45–20.18	0.252
C-HuR: positive	2.84	1.41–5.72	0.004	1.78	0.81–3.94	0.153
For high PI						
GTP: intake	0.26	0.08–0.84	0.025	0.72	0.13–4.01	0.704
GTP: 24 wks	4.38	1.03–18.6	0.045	0.93	0.13–6.86	0.944
C-HuR: positive	3.85	1.67–8.90	0.002	2.55	0.87–7.44	0.087
For COX-2						
GTP: intake	0.12	0.03–0.44	0.001	0.21	0.05–0.91	0.037
GTP: 24 wks	1.41	0.39–5.05	0.559			
C-HuR: positive	4.46	1.65–12.1	0.003	1.52	1.10–8.71	0.033
For VEGF-A						
GTP: intake	0.51	0.17–1.59	0.249			
GTP: 24 wks	2.65	0.69–10.2	0.156			
C-HuR-positive	3.85	1.67–8.90	<0.001	2.83	1.11–7.20	0.029
For HO-1						
GTP: intake	0.15	0.04–0.53	0.003	0.24	0.06–0.96	0.043
GTP: 24 wks	1.92	0.54–6.87	0.318			
C-HuR: positive	2.47	1.23–4.94	0.011	1.62	0.78–3.35	0.194

* Adjusted by cancer cell invasion

HuR; human antigen R, OR; odds ratio, CI; confidential interval, GTP; green tea polyphenol, wks; weeks, C; cytoplasmic, MVD; microvessel density, PI; proliferation index, COX; cyclooxygenase, VEGF; vascular endothelial growth factor, HO; hemeoxygenase.

All cancer-related factors examined were correlated with tumor size, angiogenesis, and tumor cell proliferation (Table 3). Similar analyses were carried out for tumors that invaded muscle, since the pathological significance of cytoplasmic HuR differs between tumors that do and do not invade muscle (Table 1 and Fig 2). COX-2 and VEGF-A expression was positively correlated with PI (r = 043, *P* = 0.038) and MVD (r = 0.47, *P* = 0.020), respectively. Furthermore, HO-1 expression was associated with MVD (r = 0.50, *P* = 0.014) and PI (r = 0.49, *P* = 0.015). No correlation was observed between tumor volume and the expression of these factors in invasive tumors; additionally, tumor volume was correlated with PI (r = 0.68, *P* < 0.001) but not MVD (r = 0.34, *P* = 0.110).

**Table 3:** Correlation between pathological characteristics and cancer-related molecules.

	COX-2	VEGF-A	HO-1
Entire; r / *P* value			
Tumor volume	0.32 / 0.025	0.39 / 0.005	0.52 / < 0.001
Microvessel density	0.60 / < 0.001	0.68 < 0.001	0.67 / < 0.001
Proliferation index	0.57 / < 0.001	0.46 / < 0.001	0.68 / < 0.001
Invasive cancer			
Tumor volume	0.20 / 0.928	0.17 / 0.430	0.35 / 0.093
Microvessel density	0.32 / 0.102	0.47 / 0.020	0.50 / 0.014
Proliferation index	0.43 / 0.038	0.15 / 0.491	0.49 / 0.015

COX; cyclooxygenase, VEGF; vascular endothelial growth factor

HO; hemeoxygenase.

## Discussion

The present study investigated changes in HuR expression following GTP intake in bladder cancer, based on the findings that HuR expression is positively associated with tumor aggressiveness—including muscle invasion—in bladder cancer patients [21]; GTP inhibits HuR expression in leukemia cells [21]; and HuR regulates the expression of various cancer-related molecules in many types of malignancy [22, 23, 30]. Our results in mice showed that cytoplasmic HuR expression was higher in cancer than in non-cancer cells, and higher in invasive as compared to non-invasive cases. These findings are consistent with trends in HuR expression reported in human bladder cancer [28]. We also confirmed for the first time in vivo that cytoplasmic HuR expression was downregulated by GTP intake. Finally, our univariate analysis showed that cytoplasmic HuR expression was associated with tumor volume, angiogenesis, tumor cell proliferation, and expression of COX-2, VEGF-A, and HO-1, which are known to induce malignant transformation of bladder cancer [27, 31]. These results indicate that our animal model is suitable for investigating the pathological roles of HuR in human bladder cancer.

In vivo and in vitro studies have demonstrated a link between HuR and malignancy. For example, HuR expression was positively associated with tumor growth in breast cancer and glioma [32, 33], angiogenesis in lung cancer [34], tumor cell proliferation in non-small cell lung cancer [30], COX-2 expression in colorectal cancer [23], VEGF-A expression in meningioma [24], and HO-1 expression in lung cancer [22]. In human bladder cancer, cytoplasmic HuR has been shown to be associated with PI, MVD, and COX-2 and VEGF-A expression [28]. Here, we found that GTP intake was also associated with these parameters, except for VEGF-A expression. GTP was previously found to inhibit tumor growth [35], angiogenesis, [36], and COX-2 [37] and HO-1 [38] expression under pathological conditions including malignancy.

**Fig 4:**
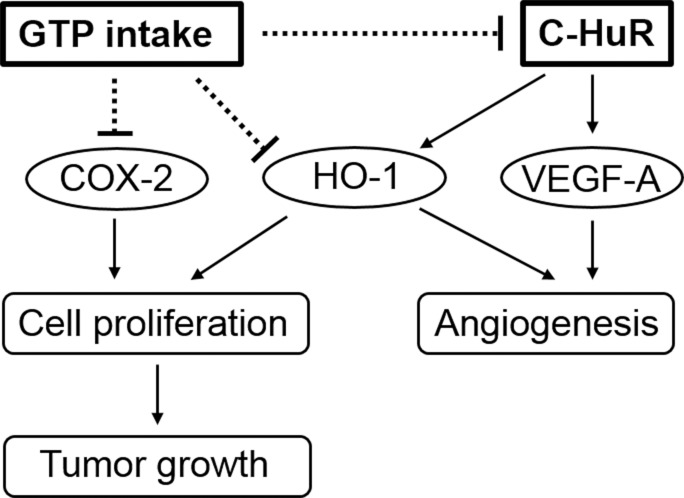
Anti-cancer mechanisms of GTP. Schematic illustration of the role of GTP intake in the modulation of malignant characteristics in bladder cancer. GTP intake suppresses the expression of COX-2 and HO-1 directly and that of HO-1 and VEGF-A indirectly via regulation of cytoplasmic HuR expression. Downregulation of COX-2 and HO-1 or HO-1 and VEGF-A leads to inhibition of cancer cell proliferation and angiogenesis, respectively, thereby suppressing tumor growth.

In the multivariate analyses, cytoplasmic expression of HuR was independently associated with that of COX-2 and VEGF-A while GTP was associated with COX-2 and HO-1 expression. Interestingly, neither cytoplasmic HuR expression nor green tea intake influenced tumor volume, angiogenesis, or tumor cell proliferation. Thus, COX-2, VEGF-A, and HO-1 expression as well as tumor volume, MVD, and PI showed significant correlations. However, in cancer tissue at 24 weeks, correlations were detected between COX-2 expression and PI, VEGF-A expression and MVD, and between HO-1 expression and MVD and PI. Tumor volume was not correlated with the expression of any cancer-related factor but was associated with PI. This suggests that changes in the malignant characteristics of bladder cancer by GTP intake are exerted via complex mechanisms involving cytoplasmic HuR (Fig 4).

A limitation of the present study was the small sample sizes, which may have resulted in a high degree of variability in the data. In fact, the effect of GTP on malignancy is controversial. For example, although GTP has been shown to induce upregulation of HO-1 in pancreatic and ovarian cell lines [39], the opposite was observed in lung cancer cells [38]. In addition, we showed that GTP intake was not associated with VEGF-A expression, whereas GTP was reported to inhibit VEGF-A in several types of cancer [40, 41]. In contrast, although we found a significant relationship between GTP intake and PI, one study reported that HuR stimulated migration and invasion but not proliferation of pancreatic ductal adenocarcinoma cells [42]. Another limitation is that we used only female mice. It has been shown that androgen and androgen receptor stimulate malignant behavior in bladder cancer cells in vitro and in vivo [43, 44]. Based on these observations, we analyzed the anti-cancer effects of GTP in female mice as accurately as possible. However, additional studies are needed in order to clarify the mechanistic basis for the anti-cancer effects of GTP.

Green tea as a preventative agent has the advantage of safety. Indeed, in a rat model of massive hepatectomy, intake of a large amount of GTP—equivalent to 100–200 times the amount consumed daily by an average individual in Japan—had no adverse effects [45]. A randomized study with a large cohort should provide more insight into the anti-cancer effects of GTP intake in bladder cancer patients.

## Conclusions

Cytoplasmic HuR expression was upregulated in BBN-induced mouse bladder cancer cells and was associated with increased muscle invasion, effects that were abrogated by GTP intake. Similarly, GTP suppressed tumor cell proliferation, angiogenesis, and expression of the cancer-related factors VEGF-A, COX-2, and HO-1. These findings suggest that GTP inhibits bladder cancer progression by direct and indirect modulation of HuR.
